# Case report: Biphasic presentation of multicystic haemorrhagic metastatic adenocarconoma of the lung

**DOI:** 10.1016/j.rmcr.2013.09.001

**Published:** 2013-09-20

**Authors:** M. Ruparel, A.A. Mohammed

**Affiliations:** Whipps Cross University Hospital, London, UK

**Keywords:** Haemorrhagic lesions, Adenocarcinoma lung, Encysted haematoma, Haemorrhagic metastases

## Abstract

Lung cancer is the one of the leading causes of death worldwide. Adenocarcinoma of the lung makes up over a quarter of all incidence of lung cancer. Multiple case reports describe haemorrhage resulting from primary or metastatic lesions affecting different organs. This case report describes an unusual presentation of a benign lung lesion that later progressed to multiple metastases with a characteristic radiological appearance. A review of prior similar reported cases is also included.

## 1 Introduction

Lung cancer is the one of the leading causes of death worldwide. Adenocarcinoma makes up approximately 25% of all cases in the UK [1]. Multiple case reports describe haemorrhage resulting from primary or metastatic lesions affecting different organs including the lungs and pleura, the adrenals, gastro-intestinal tract and brain [2], [3], [4], [5], [6], [7], [8]. However, to our knowledge, no previous reports exist describing multiple separate lesions with a cystic appearance containing blood as a result of metastatic adenocarcinoma of the lung.

## 2 Case report

**Fig. 1:**
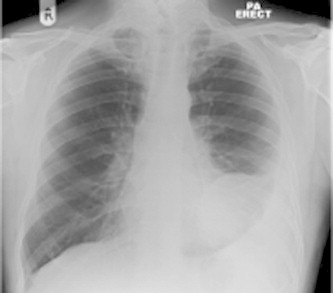
CXR on initial presentation at other district general hospital.

Mr B was a 62 year old normally fit and active man who was referred to the chest clinic for an ovoid lesion on his chest X ray. He initially presented to another district general hospital 1 year prior with fever and abnormality on chest radiograph (Fig 1). At that time the lesion was aspirated and thought be an abscess. He was treated with antibiotics and discharged. The aspirate showed no organisms and no malignant cells.

He then represented to his general practitioner ten months later who referred him to our chest clinic. He presented with a six week history of cough and some left sided chest discomfort. There was no history of sputum production. His weight and appetite were stable and there were no fevers, night sweats or haemoptysis.

He was an ex-smoker having smoked heavily in the past. There was no known asbestos exposure. He had no significant past medical or family history and he currently lived with his male partner and was still working for a leading supermarket.

On examination there was no finger clubbing or lymphadenopathy. Chest examination revealed reduced breath sounds at the left base.

**Fig. 2:**
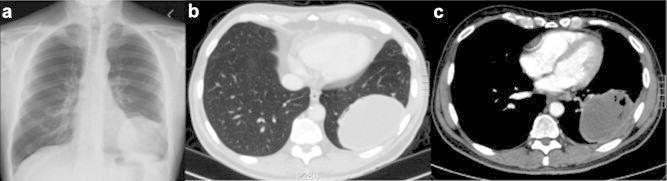
(a) CXR on presentation to our hospital (b) CT imaging -lung window (c) CT imaging -soft tissue window.

**Fig. 3:**
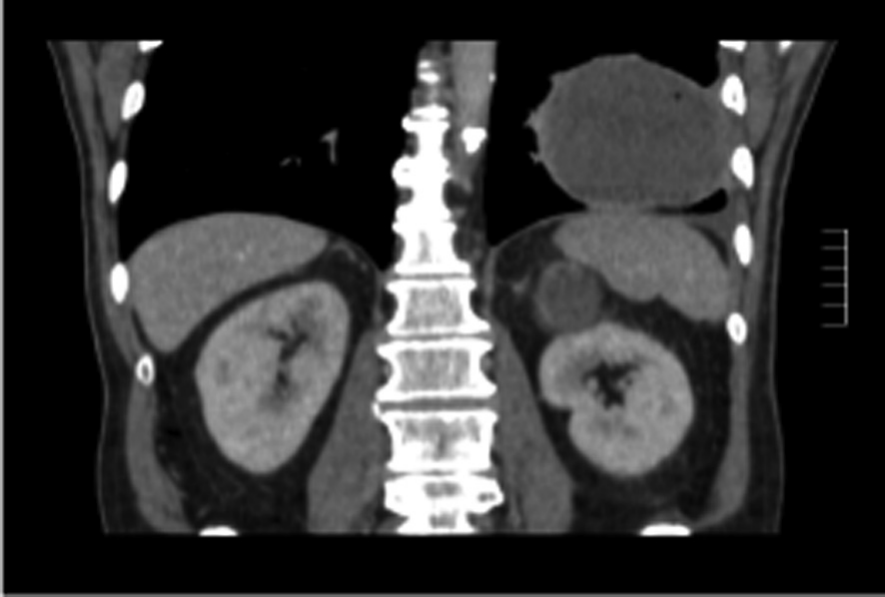
Adrenal metastasis with a blood filled appearance.

Chest radiography and CT examination (Fig 2(a),(b),(c)) revealed a 10 cm ovoid lesion in the left lower lobe adjacent to the pleura. The penetration suggested it was a fluid filled structure. A similar looking lesion was also noted in the left adrenal (Fig 3). He underwent a fibre-optic bronchoscopy. Some brown adherent material was seen at the orifice of the posterior and lateral segment of the left lower lobe. Washings and biopsies were taken. Cytology and microbiology were all negative for malignancy. He also underwent aspirations of the left lower lobe lesion and adrenal lesion. Only blood was aspirated and cytology was once again negative. HIV test was performed and was negative.

**Fig. 4:**
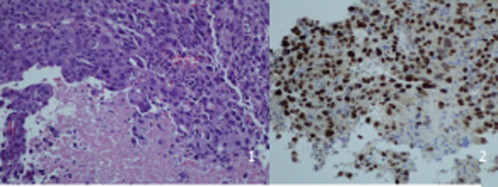
*Image 1*. Highly pleomorphic malignant epithelial cells of poorly differentiated non-small cell carcinoma. Numerous mitoses and areas of necrosis. H&E×200. *Image 2*. TTF-1 positive staining. Neoplastic cells show a moderate to strong nuclear staining with TTF-1 in favour of adenocarcinoma and lung primary. TTF-1 immunoperoxidase ×200.

**Fig. 5:**
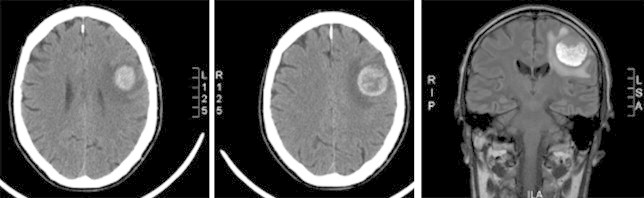
Cerebral metastasis – appearance of a blood filled cavity.

A few weeks later he developed a painful 5 cm left axillary lymph node and had this biopsied too. The biopsy confirmed metastatic adenocarcinoma suggestive of primary lung origin (Fig. 4). At this stage his performance status was still 0 and following discussion at lung MDT, it was agreed to refer him to the clinical oncologist for palliative chemotherapy. Mr B subsequently developed symptoms of headache. Imaging of the brain (CT followed by MRI) revealed a space occupying lesion in the left parietal lobe surrounded by moderate oedema (Fig 5). This was also felt to have the appearance of blood filled cystic lesions when reviewed by the radiologist. Prior to commencing chemo- or radiotherapy his case was discussed with the neurosurgeon who felt surgical intervention would be associated with high risk of mortality and can leave him with significant physical disability. Unfortunately, the day following his neurosurgical review, this lesion progressed quite rapidly causing a dense hemiplegia and slurred speech and at this stage it was felt Mr B was not fit for any active treatment. He was treated by means of symptom control and died some weeks later.

## 3 Methods

A literature search was carried out using EMBASE and MEDLINE data bases. The terms “encysted haematoma”, “haemorrhagic”, “adenocarcinoma”, “metastatic lesions” were applied to the search. Limits of English language and human reports were also applied.

## 4 Discussion

This case shows multiple cystic lesions in various organs including lung, adrenals and brain. The cytology of the blood that was aspirated from the lung and adrenal lesion simply showed blood. While initially the condition did not progress for almost a year, in the last few months, rapid progression of disease such as that seen in neoplastic disease was observed and this led to the rapid demise of our patient. The imaging initially showed a well circumscribed lesion that very much resembled an abscess. The fact that its appearance did not change much for 10 months went against a malignant diagnosis.

A report by Cabibi et al. [9], illustrates a case of mucinous cystadenocarcinoma. Here, a space is filled with extracellular mucin and with malignant cells floating in the mucin or lining and infiltrating the fibrous wall which is lined by columnar epithelium. Here they propose that such tumours start off as benign cysts or cystadenomas within the lung. These can then go on to progress to become malignant adenocarcinomas that typically express positivity for CK-7 and TTF-1. They suggest that all benign cysts in the lung have the potential to undergo malignant transformation and that early resection can be preventative against this. They also noted that only 2 such previously reported cases have been diagnosed by fine needle aspiration and that surgical resection is usually necessary to make the diagnosis. None of the cases they cited had any blood reported to be present within the lesion.

Lin and Chen [10] describe a case where a metastatic spinal epidural haematoma which was resected as it was causing a paraplegia at the level of T8. Initial imaging revealed a lesion posterior to the thecal sac from T5 to T8 with a fluid–fluid level and causing compression of the spinal cord. Immediate laminectomy was carried out and bright red blood mixed with clot gushed out on opening of the lamina. The haematoma was completely excised and sent for histology. Metastatic carcinoma was reported and the patient underwent a CT scan which showed a left hilar lung mass. Bronchial washings revealed adenocarcinoma. Their literature search revealed no other such cases and they were unclear as to the source of blood causing this haematoma. They postulated the bleeding may have resulted from the tumour itself or from epidural venous plexuses that were more friable due to the tumour itself and surrounding inflammation.

Chou et al., in 1993 [2] described what was thought to be the first case report of a spontaneous haemothorax resulting from a sub-pleural lung mass. Histology of a resected sample revealed a small perforation in the visceral pleura with tumour invasion into the pulmonary vessels and visceral pleura.

In 1980, Miller and McGreggor [11] carried out a review to evaluate haemorrage in different types of lung cancer. They found that massive haemoptysis was likely to be related to squamous cell carcinoma (SCC) and this in turn may be linked to the fact that SCC is the most likely type of tumour to be cavitating and that this process of cavitation was caused by necrosis resulting from vascular invasion by malignant cells and this in turn results in haemorrhage.

The exact reasons for the mode of this biphasic presentation seen here in this report are unclear. It is possible that the initial presentation with a febrile illness one year prior to the patient's death was due to infection within a benign lung cyst, cystadenoma or a lung abscess and that subsequent malignant transformation resulted in metastatic cancer. It is likely that the source of the blood in each lesion was from malignant invasion of blood vessels which also resulted in haematogenous spread of the tumour to various distant metastatic sites. This case report, in our view, represents the first published case of metastatic multicyctic haemorrhagic adenocarcinoma of the lung involving 3 organs (lung, adrenal and brain).

Prior to writing this article consent for publication of this case was obtained from the patient's next of kin.

## Conflict of interest

None.
